# Intraseasonal predictability of natural phytoplankton population dynamics

**DOI:** 10.1002/ece3.8234

**Published:** 2021-10-28

**Authors:** Vitul Agarwal, Chase C. James, Claire E. Widdicombe, Andrew D. Barton

**Affiliations:** ^1^ Scripps Institution of Oceanography UC San Diego La Jolla California USA; ^2^ Plymouth Marine Laboratory Plymouth UK; ^3^ Section of Ecology, Behavior and Evolution UC San Diego La Jolla California USA

**Keywords:** ecosystem forecasting, empirical dynamic modeling, functional groups, phytoplankton population dynamics, portfolio effect, predictability, Station L4

## Abstract

It is difficult to make skillful predictions about the future dynamics of marine phytoplankton populations. Here, we use a 22‐year time series of monthly average abundances for 198 phytoplankton taxa from Station L4 in the Western English Channel (1992–2014) to test whether and how aggregating phytoplankton into multi‐species assemblages can improve predictability of their temporal dynamics. Using a non‐parametric framework to assess predictability, we demonstrate that the prediction skill is significantly affected by how species data are grouped into assemblages, the presence of noise, and stochastic behavior within species. Overall, we find that predictability one month into the future increases when species are aggregated together into assemblages with more species, compared with the predictability of individual taxa. However, predictability within dinoflagellates and larger phytoplankton (>12 μm cell radius) is low overall and does not increase by aggregating similar species together. High variability in the data, due to observational error (noise) or stochasticity in population growth rates, reduces the predictability of individual species more than the predictability of assemblages. These findings show that there is greater potential for univariate prediction of species assemblages or whole‐community metrics, such as total chlorophyll or biomass, than for the individual dynamics of phytoplankton species.

## Graphical abstract

Marine phytoplankton population dynamics are inherently noisy and difficult to predict. Here, using observations of phytoplankton abundance from the English Channel, we found that the predictability of phytoplankton assemblages typically exceeds predictability for individual species and that predictability increases with assemblage size. A companion numerical model reveals that aggregation across species reduces the effect of measurement error and stochasticity in phytoplankton growth rates on predictability.

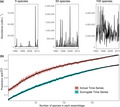

## 1 INTRODUCTION

Primary production by marine phytoplankton fuels marine ecosystems and marine fisheries (Ryther, 1969). Marine phytoplankton contribute nearly half of global net primary production (Field et al., 1998) and modulate climate by exporting carbon to ocean depths (Falkowski & Oliver, 2007). Because of the importance of phytoplankton dynamics for marine food webs, fisheries, and biogeochemical cycles, there is growing interest in making skillful future predictions of phytoplankton population and community dynamics (Anderson et al., 2016).

Phytoplankton population and community dynamics exhibit variability on rapid (daily to interseasonal) as well as longer‐term (interannual to decadal) timescales (Barton et al., 2016; Chavez et al., 2003; Chiba et al., 2012; Edwards et al., 2013; Falkowski & Oliver, 2007). These dynamics are affected by changing environmental conditions (Falkowski & Oliver, 2007; Margalef, 1978), but also by physiological (i.e., a change in organism traits without a change in genome) and evolutionary (i.e., a change in genome) responses to these changes (Collins et al., 2014; Hunter‐Cevera et al., 2014; Irwin et al., 2015; Lohbeck et al., 2012) and interactions within food webs (Di Lorenzo & Ohman, 2013; Ripa & Ives, 2003; Vasseur, 2007; Xu & Li, 2002). While environmental variations on certain spatial and temporal scales may be predictable (e.g., seasonal variations in light), the temporal and spatial trajectory of geophysical turbulence and climate dynamics are typically less predictable (Lorenz, 1963). Consequently, phytoplankton population and community dynamics that are extrinsically forced by environmental variations tend to be unpredictable. The complexity of physiological and evolutionary responses to these changes (Collins et al., 2014; Hunter‐Cevera et al., 2014; Irwin et al., 2015; Lohbeck et al., 2012), as well as non‐linear and potentially chaotic dynamics with plankton communities (Ascioti et al., 1993; Benincá et al., 2008; Giron‐Nava et al., 2017; Huisman & Weissing, 1999), adds to the difficulty of predicting plankton community dynamics. Because of the demonstrated empirical links between variations in plankton community structure, detrital flux to the benthos, and trophic efficiency and fisheries production (Stock et al., 2017), the management of living marine resources could be improved with skillful predictions of phytoplankton community structure (Hobday et al., 2016; Marshall et al., 2019; Tommasi et al., 2017).

Anomalies in phytoplankton populations (here defined as a deviation from mean conditions) rapidly decorrelate in time and space (Doney et al., 1998). For example, phytoplankton population anomalies in most of the global ocean persist for only a few weeks, on average (Kuhn et al., 2019). In contrast, midlatitude sea surface temperature and nutrient anomalies last, on average, a few months to a year or longer (Deser et al., 2003; Kuhn et al., 2019), and may have interannual persistence when subsurface anomalies are re‐exposed by deep water column mixing in subsequent years (Deser et al., 2003). Longer duration, persistent anomalies in temperature are possible due to dominant modes of climate variability and marine heatwaves, for example, in the tropical Pacific due to El Niño Southern Oscillation (Deser et al., 2010) and in the North Pacific due to the North Pacific “blob” in 2013–2015 (Bond et al., 2015). Surface temperature can, in some regions, be predicted skillfully months or even years in advance (Song et al., 2008; Stock et al., 2015; Taboada et al., 2019). Ocean surface chlorophyll and primary productivity may also be predictable, up to years in advance, though the degree of predictability varies strongly in space (Park et al., 2019; Taboada et al., 2019). Prediction skill of the physical and chemical environment, and aggregate measures of primary producers (e.g., chlorophyll and primary production) on intraseasonal and longer timescales is developing quickly (Park et al., 2019), and predictive models exist for select, influential taxa such as harmful algal bloom‐forming taxa (e.g., Anderson et al., 2016). However, ecological predictions for a broad range of plankton populations have not been conducted, and less is known about whether predictability for integrated species assemblages may differ from that of individual taxa.

In this paper, we quantified the predictability of single species and multi‐species assemblages of phytoplankton using monthly averaged phytoplankton time series data for 198 phytoplankton taxa sampled at the long‐term coastal monitoring Station L4 in the Western English Channel (50° 15'N, 4° 13'W) collected between October 1992 and December 2014. In this context, we defined an assemblage as a group of more than one species, where the abundance of the assemblage is the sum of the abundance of the individual taxa within the assemblage. Using empirical dynamic modeling (Sugihara & May, 1990), we first quantified the univariate predictability of each taxon, defined as the correlation coefficient between predicted and observed taxa abundance one month into the future. A higher correlation coefficient implies higher predictability. We then aggregated species based on several levels of taxonomic hierarchy and/or individual size, with assemblage sizes ranging from 2 to a maximum of 198 species, and assessed the assemblage predictability in comparison to the predictability of individual taxa. Our decision to directly forecast the abundance of phytoplankton assemblages (instead of aggregating the forecasted abundance of individual species) was driven by the fact that many ecological measurements, such as total chlorophyll or biomass (Barton et al., 2015), taxonomic assignations to a level of organization higher than species (e.g., diatoms), and size‐fractionated observations (Hirata et al., 2011; Marañón et al., 2012), are in practice measurements of phytoplankton assemblages.

Specifically, we ask the following: (1) “Are the dynamics of single species more or less predictable than the dynamics of assemblages of species aggregated together?”, and (2) “How does assemblage composition affect predictability?”. Our underlying hypothesis is that assemblages of species are likely to be more predictable than individual species. Such aggregated community metrics often have lower temporal variance than individual species, provided that the constituent time series are not perfectly correlated (Doak et al., 1998; Schindler et al., 2015; Vasseur & Gaedke, 2007). This process is called the “portfolio effect” and was first described in financial investment theory to improve stability by increasing portfolio size, and assuming individual investments are not perfectly correlated (Markowitz, 1952). The “portfolio effect” has been demonstrated to be effective in guiding large‐scale fisheries restoration efforts by quantifying multi‐stock dynamics (DuFour et al., 2015), as well as understanding changes in diversity–stability relationships (Lhomme & Winkel, 2002). These approaches typically use the portfolio effect as a measure of aggregate stability, such as for tracking the restoration of Chinook salmon population diversity (Yamane et al., 2018) or for monitoring soil microbial populations following perturbation (Wagg et al., 2018). The potential for using “portfolios” of phytoplankton species to improve predictability of critical, aggregate ecosystem components has not yet been evaluated. In addition, we ask the following: (3) “What factors affect the predictability of phytoplankton assemblages?”. To address this question, we developed a simple model resolving species interactions and stochasticity in a community of phytoplankton to understand the mechanisms that explain changes in predictability between individual and assemblages of taxa.

## 2 MATERIALS AND METHODS

The Materials and Methods section first describes our analysis of phytoplankton time series data from the English Channel, where we sought to quantify how predictability of aggregated time series changes with assemblage size, and thereby address questions 1 and 2 posed in the Introduction. We then describe two idealized plankton community models that help understand the factors that affect the predictability of phytoplankton assemblages (question 3).

### 2.1 Ecological data from the English Channel

**FIGURE 1:**
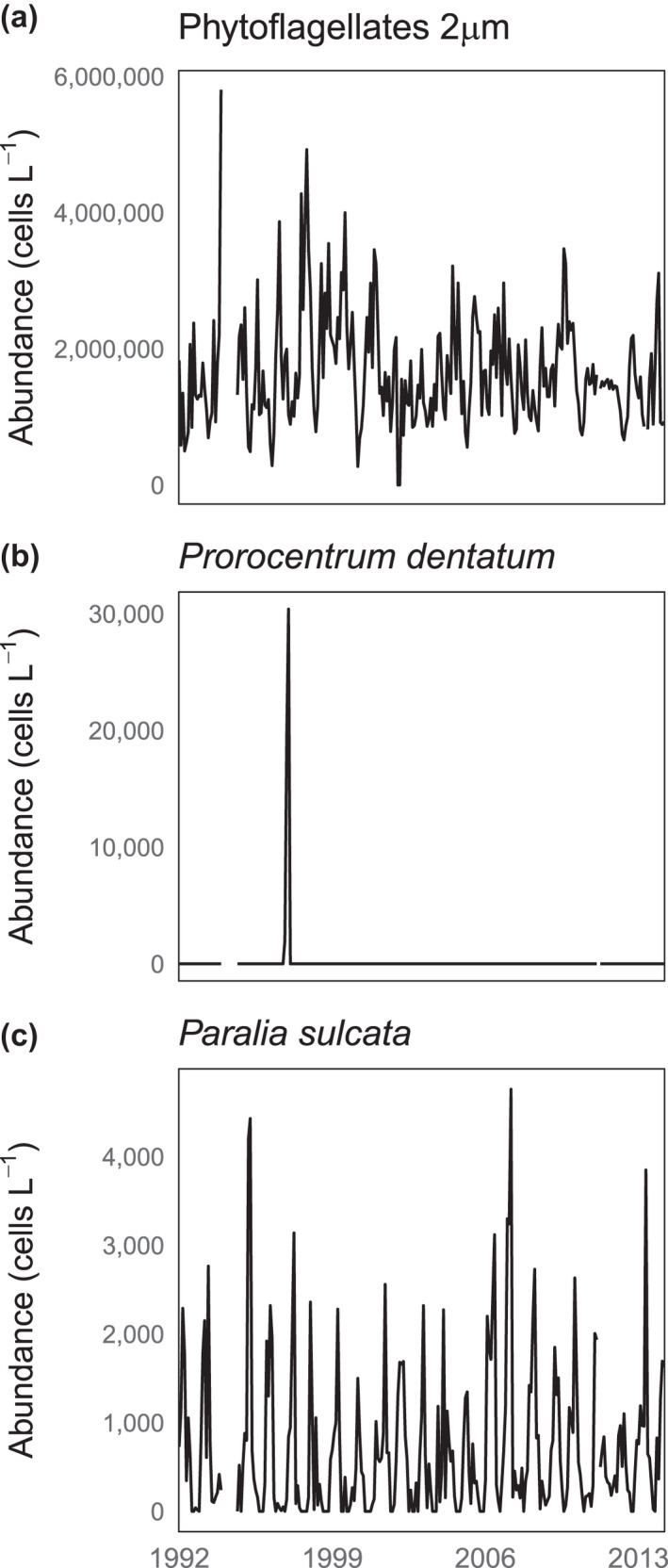
Abundance (cells L^−1^) of three different illustrative phytoplankton taxa showing various dynamics within the community: (a) Phytoflagellates 2 μm (b) *Prorocentrum dentatum* and (c) *Paralia sulcata*. There are 10 months of missing data in 22 years, as indicated by the gaps in the time series

Station L4 is a coastal marine station located approximately 10 nautical miles off Plymouth, UK, and is characterized by summer nutrient depletion with seasonal vertical and horizontal influx of nitrate into the system (Smyth et al., 2010). Total chlorophyll and functional groups of phytoplankton (e.g., diatoms) exhibit pronounced seasonal cycles at this station, but relatively small long‐term trends (Smyth et al., 2010; Widdicombe et al., 2010b). Water samples were collected on weekly basis (weather permitting) at a depth of 10 m using a 10‐L Niskin bottle, and species were identified by light microscopy using the Utermöhl technique (Widdicombe et al., 2010b). Although measurements at station L4 are conducted on a roughly weekly basis, we calculated monthly averages from the available weekly data in order to minimize gaps in the time series. Monthly averages were calculated by an arithmetic mean of all observed points for each month. The dataset includes 198 phytoplankton taxa, including well‐defined species (e.g., *Guinardia delicatula*), but also more broadly defined groups such as “Phytoflagellates 2 μm,” indicating all flagellates with a mean diameter of 2 μm (Widdicombe et al., 2010a, 2010b). In this dataset, there were a wide range of phytoplankton species and associated dynamics, from numerically abundant, generic groups such as “Phytoflagellates 2 μm” (Figure 1a) to rarely occurring *Prorocentrum dentatum* (Figure 1b) and seasonally occurring *Paralia sulcata* (Figure 1c). Each time series spans from October 1992 to December 2014, and the length is consistent for all taxa. We show the time series for these three taxa to give a sense of the range of dynamics present in the phytoplankton record.

Some of the most numerically dominant diatom species were *Leptocylindrus minimus*, *Chaetoceros socialis,* and *Pseudo*‐*nitzschia delicatissima,* whereas the most numerically dominant dinoflagellate species were *Karenia mikimotoi* and *Prorocentrum cordatum*. Further details on the phytoplankton time series, such as taxonomic classification (e.g., diatom, dinoflagellate, coccolithophore, and phytoflagellate), cell radius, mean abundance and standard deviation of abundance, can be found in Table A1 in Appendix 1.

### 2.2 Assemblage formation

We estimated the predictability of both individual time series and grouped time series created by aggregating multiple time series together (i.e., creating an assemblage of taxa). We grouped taxa together: (a) randomly, (b) within the diatom, dinoflagellate, coccolithophore and phytoflagellate functional groups, and (c) by cell size.

In the case of grouping species randomly, we selected a random subset of all taxa for each assemblage size (2, 3, 4,…, 198). In the case of taxonomic groups, we followed the same process but limited the selection to only diatoms (*n* = 130), dinoflagellates (*n* = 37), coccolithophores (*n* = 16), or phytoflagellates (*n* = 14). We excluded the time series of *Phaeocystis* for this part of the analysis as it was the only time series in its taxonomic category. In the case of grouping by cell size, we created broader categories based on approximate cell radius (<5 μm, 5–12 μm, >12 μm) and randomly sampled taxa within those categories. Each assemblage time series was an arithmetic sum of individual species cell density (cells L^−1^) at each month over time. Since some assemblages might have higher or lower prediction skill by chance, we ran 1000 trials to generate a distribution of forecast estimates for each assemblage size. Prediction estimates for each assemblage size were provided as a mean correlation value $\left(\bar{\rho }\right)$ with 95% confidence intervals (defined as ±1.96 × SE). As there are only 198 taxa that are available, it is likely that some of the 1000 trials for each assemblage size were not unique combinations of taxa (in particular, the largest assemblage sizes). To minimize selection bias, we did not make predictions on assemblages beforehand nor look at pairwise correlations within an assemblage. Each species had the same probability of being included in an assemblage.

### 2.3 Empirical dynamic modeling

We used empirical dynamic modeling (EDM) to estimate the univariate predictability of both individual and grouped time series. Briefly, EDM is a non‐parametric framework for creating predictive models of dynamic systems (Sugihara & May, 1990). EDM has been successfully applied to many problems, such as predicting fisheries recruitment (Munch et al., 2018) and demonstrating the nonlinearity of large‐scale oceanographic processes (Hsieh et al., 2005). In this study, we employed simplex projection with leave‐one‐out cross‐validation to predict phytoplankton assemblage and taxa dynamics (method described below; Sugihara & May, 1990).

Natural phytoplankton communities can be complex with large numbers of interacting species. The population dynamics of any one species can be subject to a large number of unknown variables. Takens's theorem (Takens, 1981) suggests that it is possible to reconstruct the dynamical attractor of a system with time lags of a variable of interest. This concept has been historically applied in ecology to understand the population dynamics of species in complex biological systems (Godfray & Blythe, 1990; Schaffer, 1984), including the prediction of coastal phytoplankton dynamics (McGowan et al., 2017).

Based upon state‐space reconstruction using Takens's theorem (Deyle & Sugihara, 2011), we focused on the univariate prediction skill of individual or aggregated time series—to test how well an individual time series can predict itself. This is an equation‐free approach that allows us to recover mechanistic relationships in the ecosystem (DeAngelis & Yurek, 2015; Ye et al., 2015). Univariate attractors are constructed from lagged embeddings of the variable of interest. The number of lags is determined by *E*, the embedding dimension. A detailed discussion on embedding dimensions can be found in Godfray and Blythe (1990). Univariate attractors can then be represented as a set of coordinates in E‐dimensional space $\left(x_{t},x_{t‐1},x_{t‐2}\ldots x_{t‐\left(E‐1\right)}\right)$, where $x_{t}$ is the value of a variable of interest, *x* at time *t*, and $x_{t‐1}$ is its value at time *t*−1, etc. We selected for the optimal embedding dimension (i.e., the *E* with the highest prediction skill *ρ*) from 1 to 10 for each phytoplankton time series, whether for individual species abundance or for aggregate assemblages. Following this reconstruction in E‐dimensions, we then look at the nearest neighbors of every point and track their movement in time. Future predictions are the weighted average of the trajectory of the nearest neighbors using leave‐one‐out cross‐validation. Further details on the method outlined here can be found in Sugihara and May (1990). Empirical dynamic modeling is one of many different approaches to make short‐term predictions using time series embeddings (Casdagli, 1989; Farmer & Sidorowich, 1987).

Prediction estimates are given by *ρ* (0 ≤ *ρ* ≤ 1), which is the Pearson correlation coefficient between observed data and the predicted values based on time series reconstruction. A higher *ρ* means that predictions a month in advance are closer to observations, whereas lower *ρ* values mean the predictions are less accurate. We elected to use ρ as a metric of prediction skill, as opposed to other possible metrics (Kim & Kim, 2016), because of its simplicity and widespread use in empirical dynamical modeling studies (e.g., Chang et al., 2017). We next assess whether predictability exceeds what would be expected from seasonal ecological changes.

Phytoplankton in natural ecosystems often respond to seasonal environmental and biological changes. Therefore, we describe a method for testing whether the predictability we estimate within each time series exceeds what is expected from seasonality alone. Here, we implemented a surrogate test for each time series prediction. Surrogate time series were created in a series of steps: (i) calculate the climatological seasonal cycle for each taxon or assemblage (i.e., average all the data from all Januarys in the time series to calculate the average January phytoplankton abundance), (ii) calculate the residuals, or anomalies, by subtracting the seasonal cycle from each time series, (iii) shuffle the time series of residuals, and (iv) add the shuffled residuals back to the repeating climatological seasonal cycle. The resulting surrogate time series removes the ecological dynamics within the time series but retains the seasonal cycle. We then calculated a *ρ* value using the simplex methodology described above for this surrogate time series to assess the predictability of the seasonality. For each time series (1000 trials each of assemblage size 1, 2, 3, … maximum assemblage size), we created 100 surrogate time series. A direct comparison of *ρ* between a time series and its associated surrogates then indicates the strength of our model in predicting actual ecosystem dynamics over seasonal forcing. All analyses were conducted with the “rEDM” package (v1.2.3; https://github.com/SugiharaLab/rEDM) in R (R Core Team, 2020). All the plots were created using the R package “ggplot2” (Wickham, 2016).

### 2.4 Noise modeling

We created a simple model to test how noise added to a time series, for example as measurement error, influences predictability. In the model, the abundance of each species through time ($x_{i,t}$) is a sine function with a species‐specific phase shift and time‐varying noise added:

(1) $$x_{i,t}=A\cdot \text{sin}\left(\omega t+\phi \right)+A+\gamma^{\text{obs}}\cdot \varepsilon_{i,t}^{\text{obs}}+\beta_{i}^{\text{obs}}$$

where $x_{i,t}$ is the abundance of taxon $x_{i}$ at time $t\left({\text{cells L}}^{‐1}\right)$, A is the amplitude $\left(\text{unitless}\right)$, ω is the frequency of oscillation, ϕ is the phase shift $\left(\text{rad}\right)$, $\gamma^{\text{obs}}$ is the scaling factor of noise $\left(\text{unitless}\right)$, $\varepsilon_{i,t}^{\text{obs}}$ is the observational error $\left({\text{cells L}}^{‐1}\right)$, and $\beta^{\text{obs}}$ is an offset $\left({\text{cells L}}^{‐1}\right)$ to ensure that $x_{i,t}\geq 0.$

$$\beta_{i}^{\text{obs}}=\left|\text{min}\left(\gamma^{\text{obs}}\cdot \varepsilon_{i,t}^{\text{obs}}\right)\right|$$

$$\varepsilon_{i,t}^{\text{obs}}∼N\left(\mu =0,\sigma^{2}=1\right)$$

The amount of observational error in the time series ($\varepsilon_{i,t}^{\text{obs}}$) was selected from normally distributed noise and modulated by $\gamma^{\text{obs}}$. Observational error refers to error occurring from the sampling process. In this example, the model resolution is monthly, and we add noise to each monthly time step. We tested the predictability of 100 species in assemblages (of 1, 2, 3…100 species) with three levels of noise (γ=0,0.5and1). The phase shift (ϕ) allowed for some variability in dynamics across the model species. The values for each of the parameters can be found in Table 1. Sample time series from the model can be found in Figure S1.

**TABLE 1:** List of model parameters, units, and values used in the noise model

Symbol	Parameters	Units	Value(s)
*t*	Time step	months	1
*x_i_*	Abundance of species i	cells L^−1^	
*A*	Amplitude of oscillation	–	1
ϕ	Phase shift	rad	‐1≤ϕ≤1
ω	Frequency of oscillation	rad month^−1^	$\frac{2\pi }{12}$
$\gamma^{\text{obs}}$	Scaling factor for noise	–	0, 0.5 (low) and 1 (high)
$\varepsilon_{i,t}^{\text{obs}}$	Observational error for species *i*	cells L^−1^	$∼N\left(\mu =0,\;\sigma^{2}=1\right)$
$\beta_{i}^{\text{obs}}$	Offset to ensure $x_{i}\geq 0$	cells L^−1^	$\left|\text{min}\left(\gamma^{\text{obs}}\cdot \varepsilon_{i,t}^{\text{obs}}\right)\right|$

### 2.5 Phytoplankton community modeling

We next describe an idealized model of an interacting phytoplankton community that we used sequentially to test how inter‐species interactions and stochasticity in vital rates influence predictability for individual and multiple model taxa. The model is based on the Lotka‐Volterra competition equations (Lotka, 1920). The abundance of species i, $x_{i}\left({\text{cells L}}^{‐1}\right)$, is controlled by the realized population growth rate $r_{i,t}\left({\text{day}}^{‐1}\right)$, the carrying capacity $K_{i,t}\left({\text{cells L}}^{‐1}\right)$, and the sum of interactions ($\alpha_{i,j};\text{unitless}$) with other species ($x_{j}$):

(2) $$\frac{dx_{i}}{dt}=r_{i,t}x_{i}\left(1‐\frac{\sum_{j=1}^{N}\alpha_{i,j}x_{j}}{K_{i,t}}\right)$$

The realized population growth rate and carrying capacity for each species vary through time and are explained below. We allowed each species to interact with only one‐fourth the total number of species in the ecosystem, and interaction strength was regulated by α. The interaction strength between any two species $\alpha_{i,j}$ was randomly selected from a uniform distribution with limits ±α and varied on a monthly basis. We created two treatments for interaction strength: (i) α=0 for no interactions between species and (ii) α=0.25 for strong interactions between species. We limited the number of interacting species to one‐fourth the total in order to simulate a community where many but not all species interact directly. Since interactions could be both positive and negative, every species had a minimum abundance of $1\times 10^{‐10}{\text{cells L}}^{‐1}$ through time to prevent extinction.

The carrying capacity of each species ($K_{i,t})$ was controlled by the seasonal cycle without the addition of noise (Equation 3):

(3) $$K_{i,t}=K\cdot \left(A\cdot \text{sin}\left(\omega t\right)+\delta \right)$$

δ=A+0.1

The scaling parameter *K* was constant and equal for every species. We added a small value δ to ensure that $K_{i,t}>0$. Each species had a basal physiological growth rate $\mu \left({\text{day}}^{‐1}\right)$ selected from 0.8 to 1.2. To introduce process noise into the system (i.e., noise added to the key organism trait in the model), we added randomly generated values of growth rate $\varepsilon_{i}^{p}\left({\text{day}}^{‐1}\right)$ to the seasonal cycle of each species. The values were selected from a normal distribution$∼N\left(\text{mean}=0,\;\sigma^{2}=1\right)$. We chose to add noise on monthly timescales, assuming that environmental processes that influence growth rates (such as sea surface temperature anomalies) persist for weeks to months (Kuhn et al., 2019). The realized population growth rate for each species ($r_{i}$) at time *t* was a function of the physiological growth rate (μ), the seasonal cycle, and total amount of process noise $\left(\gamma^{p}\cdot \varepsilon_{i,t}^{p}\right)$:

(4) $$r_{i}=\mu_{i}\cdot \left(A_{i}\cdot \sin\left(\omega t\right)+\mu_{i}+\gamma^{p}\cdot \varepsilon_{i,t}^{p}+\beta_{i}^{p}\right)$$

$$\beta_{i}^{p}=\left|\text{min}\left(\gamma^{p}\cdot \varepsilon_{i,t}^{p}\right)\right|$$

The parameter $\gamma^{p}$ was determined at the start of each experiment. We had two treatments for adding process noise: (i) $\gamma^{p}=0$ for no process noise and (ii) $\gamma^{p}=1$ for high process noise. $\beta^{p}\left({\text{day}}^{‐1}\right)$ is an offset to ensure that $r_{i}\geq 0$ after the addition of process noise.

We simulated 20 years of data from our model with a 6‐h time step. We aggregated the 6‐hourly data to monthly averages to maintain our timescales of prediction and keep our model comparable to L4 monthly averaged data. Our goal was to check the effects of interaction strength (α) and level of process noise ($\gamma^{p})$ on prediction skill (*ρ*) across different assemblage sizes in the model ecosystem. Values for each of the parameters in the model can be found in Table 2. Sample time series from the model can be found in Figure S2.

**TABLE 2:** List of model parameters, units, and values used in the phytoplankton community model

Symbol	Parameters	Units	Value(s)
*t*	Time step	hours	6
*x_i_*	Abundance of species *i*	cells L^−1^	
*A*	Amplitude of oscillation	–	1
ω	Frequency of oscillation	rad day^−1^	$\frac{2\pi }{360}$
α	Limits of interaction strength	–	$0\;\left(\text{no interactions}\right)$ 0.25 (strong interactions)
$\alpha_{i,j}$	Interaction strength between two species	–	$‐\alpha <\alpha_{i,j}<\alpha$
μ	Physiological growth rate	day^−1^	0.8≤μ≤1.2
K	Carrying capacity scaling factor	cells L^−1^	30,000
$K_{i}$	Effective carrying capacity for species *i*	cells L^−1^	
δ	Offset to ensure $K_{i}>0$	–	A+0.1
$r_{i}$	Realized growth rate for species *i*	day^−1^	
$\gamma^{p}$	Scaling factor for noise	–	0 (no noise), 1(high noise)
$\varepsilon_{i,t}^{p}$	Process noise for species *i*	day^−1^	$∼N\left(\mu =0,\sigma^{2}=1\right)$
$\beta_{i}^{p}$	Offset to ensure $r_{i}\geq 0$	day^−1^	$\left|\text{min}\left(\gamma^{p}\cdot \varepsilon_{i,t}^{p}\right)\right|$

## 3 RESULTS AND DISCUSSION

### 3.1 Are single species more or less predictable than assemblages of species?

#### 3.1.1 How does assemblage size affect predictability?

**FIGURE 2:**
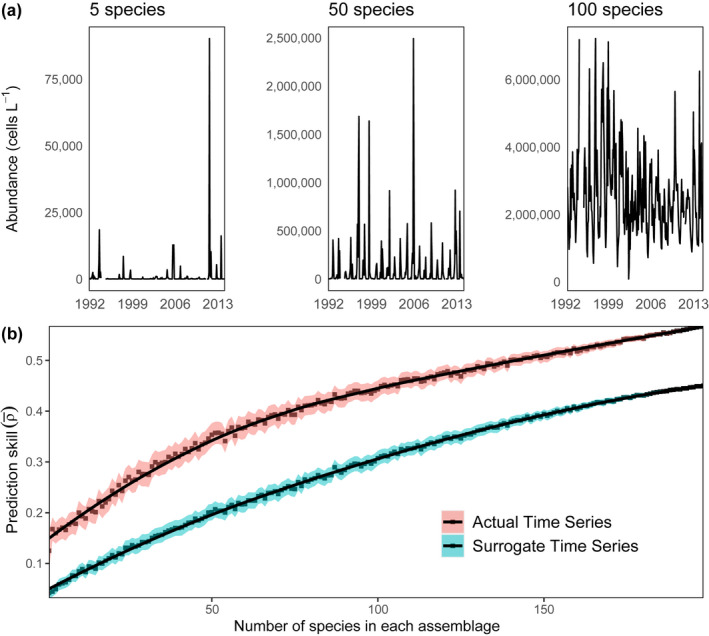
(a) Abundance (cells L^−1^) of 5, 50, and 100 randomly grouped species from October 1992 to December 2014, and (b) mean prediction skill ($\bar{\rho }$) for assemblages of species ranging in size from 1 to 198 species in the assemblage (red) and their seasonal surrogates (blue). Each point is the mean of 1000 trials, and the black lines represent local regression fits for both sets of data (actual time series and surrogates). The shaded regions are 95% confidence intervals (defined as ±1.96×SE). The confidence intervals narrow with increasing assemblage size because the number of distinct assemblages of species decreases

Predictability increased with the number of aggregated taxa, regardless of taxonomy, and the predictability exceeded what would be expected from seasonality alone. While individual or assemblages with few species tend to exhibit noisy dynamics, larger aggregated assemblage sizes tended to exhibit smoother, more repeating annual cycles of abundance (Figure 2a; this figure shows three illustrative abundance time series with 5, 50, and 100 species added together). In this case, species were aggregated randomly with no regard for their taxonomic grouping. The average coefficient of variation for each of the newly formed assemblages decreases with increasing assemblage size (Figure S3). Prediction skill increased with assemblage size for the actual time series and surrogates (Figure 2b). However, $\bar{\rho }$ was greater for the actual time series than for the seasonal surrogates, suggesting that the predictability is not simply a function of seasonality. The rate of increase in predictability with increasing assemblage size saturated for large assemblage sizes, possibly pointing to a maximum level of prediction for assemblages of species in this system. We hypothesize that the existence of a maximum could be tied to the presence of noise in our data, originating from a range of sources such as observational error or stochastic environmental influence, which control the overall limits of being able to predict phytoplankton assemblage dynamics. Time series length might also play an important role in determining the maximum level of predictability of an assemblage or species, as it has been shown that the number of observations can have an impact on the predictability of plankton dynamics (Giron‐Nava et al., 2017).

One implication of this result using univariate methods is that while individual species may not be highly predictable on average, many species aggregated together can be much more predictable. This result appears consistent with empirical studies from aquatic and marine settings (Schindler et al., 2015) as well as theory (Koellner & Schmitz, 2006). For example, experimental acidification of lakes has found that species composition changes markedly while total biomass may not (Frost et al., 1995; Schindler, 1990) and that experimental nutrient enrichment across multiple lakes produced contrasting responses in community structure but relatively consistent increases in total biomass (Cottingham & Carpenter, 1998). Mutshinda et al. (2016) found that the total biomass dynamics of diatoms and dinoflagellates at the L4 station in the English Channel were distinct from one another and tied to environmental variations, but that the biomass of individual species within each assemblage was typically less tied to environmental conditions.

Most ecosystems have a mix of abundant and rare species, and some individual species have higher individual predictability than others. As assemblage size increases, the likelihood that the few abundant and/or more predictable species will be included in the assemblage increases. We tested whether the increase in assemblage predictability with assemblage size (Figure 2) could be attributed to the inclusion of more abundant and/or predictable taxa by repeating the analysis in Figure 2 but excluding: (a) the top 25% of most numerically abundant taxa, defined as the time series with highest mean abundance through time (Figure S4) and (b) the top 25% of most predictable taxa, defined as the time series with the highest univariate predictability through time (Figure S5). Even when excluding the most abundant or predictable species, we found that predictability increased with assemblage size.

### 3.2 What factors affect the predictability of phytoplankton assemblages?

#### 3.2.1 The effect of noise on model time series predictions

**FIGURE 3:**
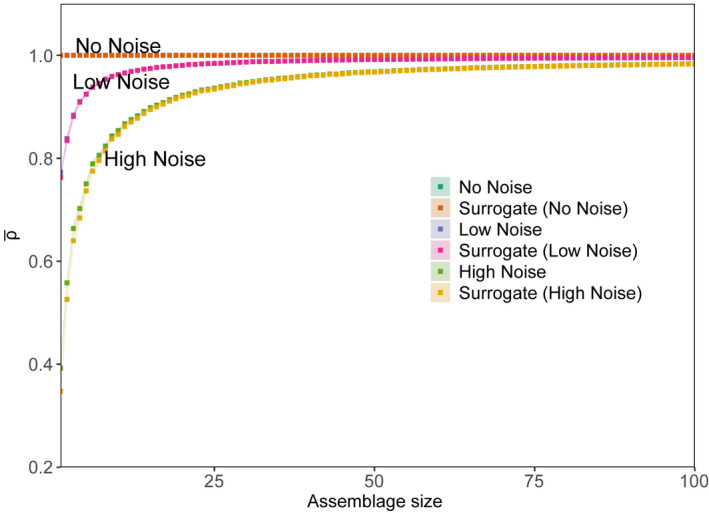
Mean prediction skill ($\bar{\rho }$) by assemblage size for the simple model exploring how measurement noise affects predictability (Equation 1), with three levels of noise (No noise, Low and High noise levels). Each point is the mean of 100 trials at each assemblage size. The shaded regions are 95% confidence intervals (defined as ±1.96×SE). The model predictions overlap with the surrogate predictions because there are no dynamics beyond seasonality in the model

Using the simple model where species differed only in the timing of their seasonal blooms and noise added to the time series (Equation 1, Section 2.4), we found that increasing the level of noise decreases the predictability of model populations (Figure 3). In the case where no noise is added to the repeating seasonal cycles of abundance, the system had perfect predictability ($\bar{\rho }$ = 1) and increasing the assemblage size did not change predictability. As the noise increases, the maximum prediction skill decreases, and this reduction is particularly evident for the individual time series (assemblage size = 1) (Figure 3). In the case of low and high noise ($\gamma^{\text{obs}}$ of 0.5 and 1, respectively), prediction skill increased with assemblage size, as for the L4 times series data (Figure 2). The aggregation of multiple time series amplified the seasonal cycles by diluting the effect of noise. This is also why the surrogate time series were as predictable as the model time series. These results suggest that noise from a range of sources, such as differences in sampling method and high variance among replicate samples, tends to decrease predictability. The larger the amount of noise added, the larger the assemblage size must be to achieve high predictability. Given that there will always be a range of sources of measurement error in phytoplankton time series such as at L4 (see Barton et al., 2020), this model result suggests that aggregating across multiple species may be a desirable strategy for making predictions.

#### 3.2.2 Simulated species interactions and stochasticity

**FIGURE 4:**
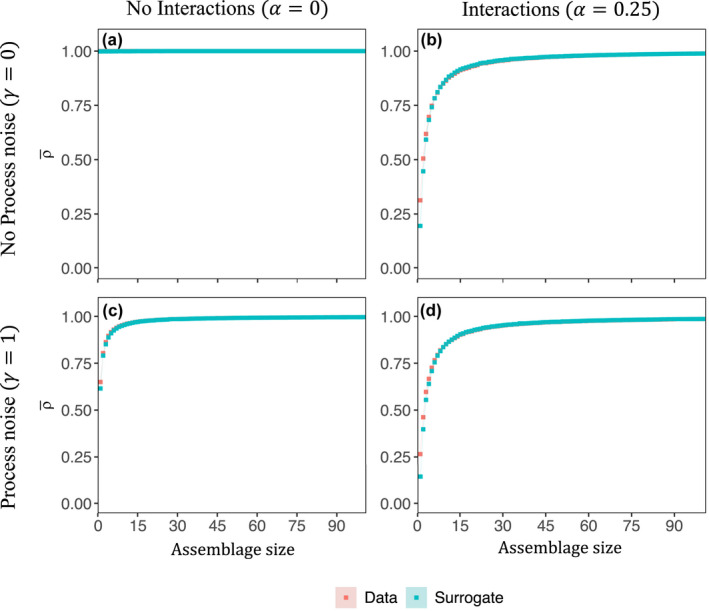
Mean prediction skill ($\bar{\rho }$) by assemblage size for the model resolving variations in growth rate and carrying capacity (Equations (2), (3), (4)) with (right column) and without inter‐species interactions (left column). The model was run without stochasticity in growth rates (e.g., process noise; top row) and with stochasticity in growth rates (bottom row). Each point is the mean of 100 trials at each assemblage size. The shaded regions are 95% confidence intervals (defined as ±1.96×SE). The difference between model data (red) and surrogates (blue) rapidly decreased with increasing assemblage size.

Using a simple ecological model that resolves species interactions and stochastic variations in growth rates ((2), (3), (4), Section 2.5, Figure S2), we examined how interaction strength and stochastic behavior in growth rates influence predictability. In the case where species did not interact $\left(\alpha =0\right)$ and there was no stochasticity ($\gamma^{p}=0)$ in growth rates, the predictability of all assemblages was perfect ($\bar{\rho }$ = 1) and did not increase with assemblage size (Figure 4a). In the case where there was no stochasticity ($\gamma^{p}=0)$ in growth rates, but species interacted (α=0.25, Figure 4b), the predictability of individual species was lower than the predictability of aggregated assemblages. Prediction skill increased with assemblage size and exceeded what would be expected from seasonality alone. In the case with stochasticity in growth rates ($\gamma^{p}=1)$ but no interactions between species (α=0, Figure 4c), the predictability of individual species was lower than the predictability of assemblages and increased with assemblage size. This predictability was also greater than what would be expected from seasonality. We found a similar result in the case with both stochasticity and species interactions ($\gamma^{p}=1,\;\alpha =0.25;$ Figure 4d). Thus, when stochastic variations in growth rate are uncorrelated across species, the dynamics of individual species may be more difficult to predict than assemblages containing many species. The dynamics of individual species are also more difficult to predict in the presence of inter‐species interactions. Because there are many possible factors that influence the growth of phytoplankton in real systems that may be difficult to resolve, and because the interactions between species are in many cases difficult to assess, our results suggest under these conditions, predicting single species will be difficult while prediction of assemblages of species may be more skillful.

### 3.3 How does assemblage composition affect predictability?

**FIGURE 5:**
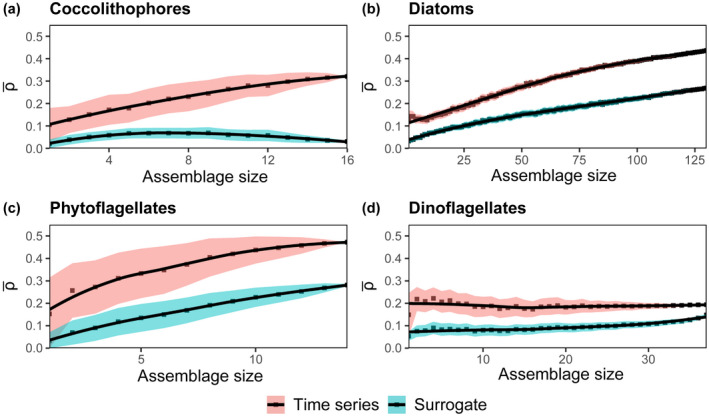
Mean prediction skill ($\bar{\rho }$) of time series (red) and seasonal surrogates (blue) for (a) coccolithophores (*n* = 16), (b) diatoms (*n* = 130), (c) phytoflagellates (*n* = *14*), and (d) dinoflagellates (*n* = *37*). Each point is the mean of 1000 trials, and the black lines represent local regression fits for both sets of data (actual time series and surrogates). The shaded regions are 95% confidence intervals (defined as ±1.96×SE).

**FIGURE 6:**
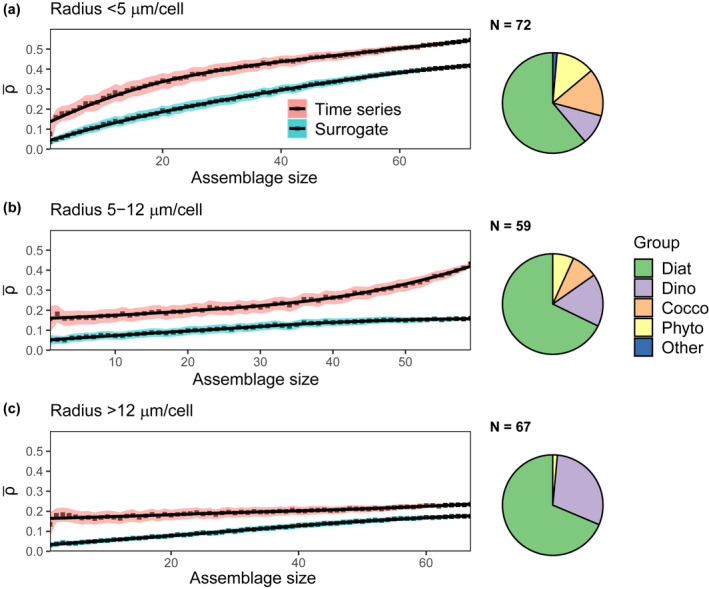
Mean prediction skill ($\bar{\rho }$) of time series (red) and their seasonal surrogates (blue) for cells with radius (a) <5 μm (*n* = 68), (b) 5–12 μm (*n* = 63), and (c) >12 μm (*n* = 67). Each point is the mean of 1000 trials, and the black lines represent local regression fits for both sets of data (actual time series and surrogates). The shaded regions are 95% confidence intervals (defined as ±1.96×SE). Pie graphs at right provide the relative diversity of functional groups within each size‐based category (Diat, diatoms; Dino, dinoflagellates; Cocco, coccolithophores; Phyto, phytoflagellates; Other, *Phaeocystis*)

We also explored whether predictability varied across functional groups of species (e.g., diatoms, dinoflagellates, coccolithophores, and phytoflagellates) and across cell size (Figures 5 and 6). We first analyzed the predictability of diatoms, dinoflagellates, coccolithophores, and phytoflagellates, as these functional groups are well‐represented at the L4 station, with 130, 37, 16, and 14 taxa, respectively. In many applications, taxonomically similar species are analyzed (Widdicombe et al., 2010b) or modeled (Le Quere et al., 2005) collectively rather than on a species level, even though species within functional groups in many cases have different traits (Edwards et al., 2012; Marañón et al., 2013) and ecological dynamics (Edwards et al., 2013; Mutshinda et al., 2016). We also separated taxa measured at L4 into three size classes: small (<5 μm), medium (5–12 μm), and large (>12 μm). The size cutoffs are arbitrary but designed so that each assemblage has a roughly equal number of taxa. Like functional groups, in many cases phytoplankton of similar size are aggregated together in field measurements or satellite algorithms (Hirata et al., 2011). Cell size constrains many important organism traits, such as growth rate and nutrient affinity (Edwards et al., 2012; Marañón et al., 2013), as well as predator–prey interactions (Hansen et al., 1994, 1997) and therefore may provide an additional way of grouping phytoplankton, and drawing out any differences in predictability that could arise as a result of assemblage composition. For each taxonomy or size‐based assemblage analyzed, we found that the coefficient of variation decreased, on average, over the multiple possible groupings with increasing assemblage size (Figure S3).

#### 3.3.1 Taxonomy

Mean prediction skill $\left(\bar{\rho }\right)$ increased with assemblage size for diatoms, coccolithophores, and phytoflagellates (Figure 5a–c). Unlike other functional groups, the predictability of dinoflagellates did not increase with assemblage size (Figure 5d). For all the functional groups, there was a clear difference between the predictive skill of time series and predictions based on seasonality alone. Dinoflagellates also had lower maximum predictability at high assemblage size than other groups (lower $\bar{\rho }$ in Figure 5d compared to others). Why do dinoflagellates apparently differ from other groups in this regard?

Dinoflagellates are a morphologically and physiologically diverse functional group of phytoplankton (Brandenburg et al., 2018; Hackett et al., 2004; Smayda & Reynolds, 2003). They exhibit not just a range of morphology and size‐constrained traits, but also large variations in trophic mode, motility, production of allelopathic chemicals, and other traits (Smayda, 1997; Stoecker et al., 2017). Since predictability did not increase with assemblage size, we believe that dinoflagellate species have dynamics that arise from different processes from each other (i.e., aggregated data are not indicative of a common process on a larger ecological scale). Unlike dinoflagellates, the predictability of both diatoms and coccolithophores increased with group size (Figure 5a and b). The differing performance of aggregated coccolithophores, diatoms, dinoflagellates, and phytoflagellates suggests that increased predictability for phytoplankton assemblages is dependent on the choice of taxa in each assemblage. An implication of the lower performance of certain assemblages is that field measurement programs should focus efforts on identifying to species level those phytoplankton, such as the dinoflagellates, for which predictability does not increase with group size. In contrast, for functional groups such as diatoms, a coarser level of identification (i.e., to genera) would not limit the utility of the data for making predictions.

#### 3.3.2 Cell size

**FIGURE 7:**
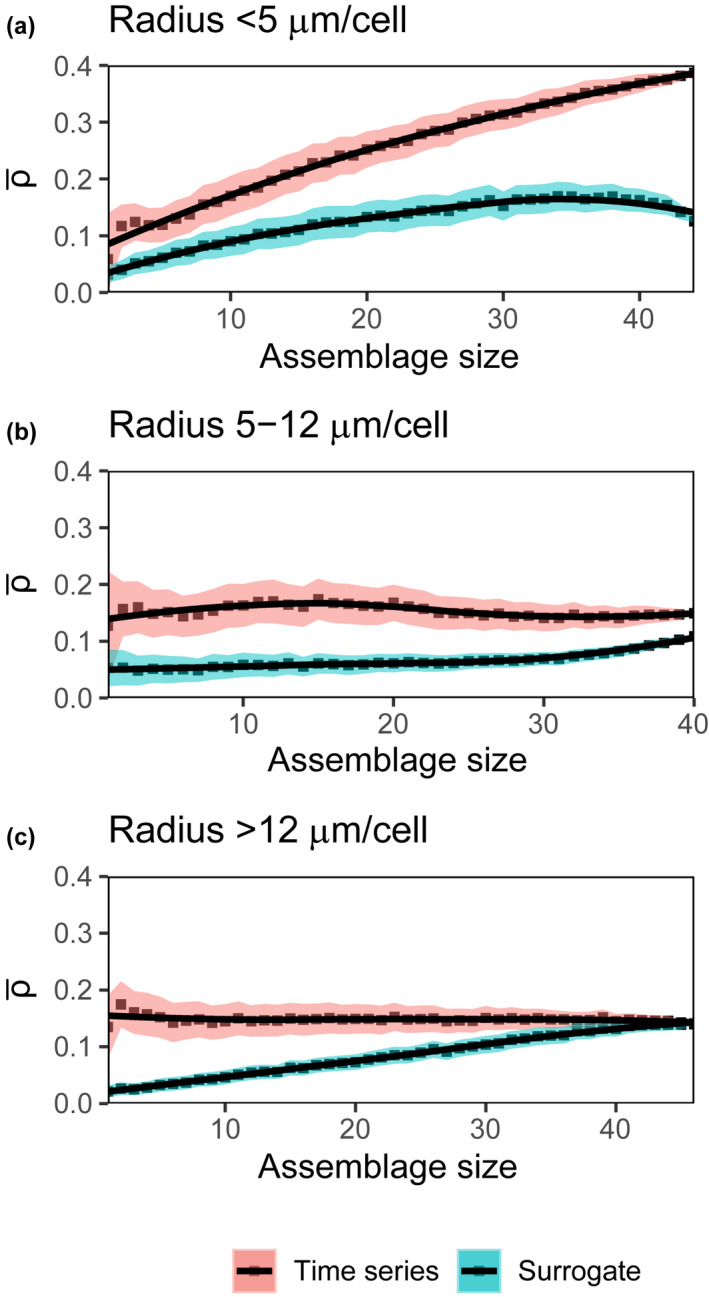
Mean prediction skill ($\bar{\rho }$) of time series (red) and their seasonal surrogates (blue) for only diatoms of the following radius: (a) <5 μm (*n* = 44), (b) 5–12 μm (*n* = 40), and (c) >12 μm (*n* = 46). Each point is the mean of 1000 trials, and the black lines represent local regression fits for both sets of data (actual time series and surrogates). The shaded regions are 95% confidence intervals (defined as ±1.96×SE)

Next, we describe how predictability $\left(\bar{\rho }\right)$ changed across three size bins (<5 μm, 5–12 μm, and >12 μm). For cells in the <5 μm size range, predictability increased with assemblage size (Figure 6a). The increase in predictability with assemblage size was minor for cells in the 5–12 μm size range (Figure 6b) and negligible for the largest cells (Figure 6c). The predictability at maximum assemblage size was lower for the larger phytoplankton size class compared with the smallest phytoplankton size class (comparing $\bar{\rho }$ across Figure 6a–c). A similar result was apparent when we looked only within the diatoms (Figure 7): predictability increased with assemblage size for the smallest diatoms (Figure 7a) but did not change for the larger diatoms (Figure 7b and c). The relative insensitivity of prediction skill to assemblage size for this size class might be the result of episodic bloom events which are common among larger phytoplankton (Irigoien et al., 2004) and can be difficult to predict.

There are several implications of this finding. First, because increasing assemblage size does not lead to increased prediction strength in large phytoplankton (including among diatoms; Figures 6c and 7c), field measurement programs ideally should continue to measure and identify all species in this size range individually. In order to predict the dynamics of large phytoplankton, each species should be considered independently of each other as it is more important to retain species‐specific information. Better predictions of large phytoplankton may be achieved by carefully evaluating external drivers and important variables for every species individually. In contrast, for the smallest phytoplankton, skillful predictions of integrated biomass of small phytoplankton might be achieved by measuring a few tens instead of all species (Figures 6a and 7a). This suggests that numerical models used for ecological prediction and forecasting, which typically resolve a few to as many as a hundred phytoplankton, may need to resolve even more biodiversity among the larger size classes (Dutkiewicz et al., 2015; Follows et al., 2007). Similarly, for field monitoring programs designed to detect (Widdicombe et al., 2010b) and forecast changes in marine ecosystems, an ideal sample design might focus taxonomic identification time on the larger taxa but allow some of the smaller, harder to identify taxa to be aggregated together. This could be done using automated or supervised quantification of machine classifiers (Orenstein et al., 2020) that are preferentially trained on larger taxa. The use of flow cytometry or similar flow‐through counting of smaller cells might also make monitoring programs more efficient without reducing the utility of data for forecasting changes in marine ecosystems.

### 3.4 Study limitations and future directions

Here, we discuss several methodological choices that influence our results. First, we chose to forecast using aggregated data across multiple species, as this is a common feature of many oceanographic and ecological observations. However, in certain cases and systems, aggregating the forecasts of individual species may be more appropriate, for example, if a particular species has disproportionate impacts, such as for harmful algal bloom taxa (Trainer et al., 2012). Second, we used all raw time series from the L4 database which varied in taxonomic resolution (e.g., size‐fractionated class like “Phytoflagellates 2 μm” and genera like *Chaetoceros* spp.). It is likely that the actual predictability of some assemblages might either increase or decrease due to the inclusion of time series with a lower taxonomic resolution. Future studies may consider quantifying this effect by differentiating predictability across different levels of aggregation. Finally, we chose to use monthly data for our analyses, though considerable phytoplankton dynamics exist at higher frequency. This choice reflected practical considerations regarding data availability, and we were unable to detect any high‐frequency dynamics as a result; however, future studies should address how data collection frequency influences predictability.

Anomalies in phytoplankton populations rapidly decorrelate in time and space (Kuhn et al., 2019). Our study was limited in spatial resolution because Station L4 is a fixed sampling location. Because Station L4 is a coastal site, an increase in the spatial resolution of phytoplankton time series might capture more variable environmental conditions and associated dynamics for both phytoplankton species and assemblages of species. Similarly, the temporal resolution of our study was limited by data availability. Our forecast distance of a month reflects a compromise between data resolution and the likely window of skillful future predictions using our methodology. Predictions at timescales shorter than a month were not possible given the data availability (we used monthly resolved time series), and predictions at timescales longer than a month are difficult because phytoplankton population dynamics rapidly decorrelate in time. However, future studies could consider systematically testing the effect of temporal resolution on forecast skill for phytoplankton species *vs* assemblages, as well as forecasting beyond one month. Understanding the effects of temporal resolution on forecast skill could answer questions about the optimal frequency of field sampling for phytoplankton populations and the appropriate lags to consider while predicting their dynamics.

There are various additional approaches that can be used to maximize the predictability of any time series, but here we used a univariate simplex approach, instead of bringing in other ecological or environmental variables to improve prediction. One such approach would be multiview embeddings (Ye & Sugihara, 2016). Multiview embeddings are a multivariate approach that leverage information in causally related variables to increase the predictability of any individual time series. This typically relies on identifying important variables for every taxon or iteratively testing multiple variable combinations for each time series. In our study, the availability of 22 years of data and 198 individual time series allowed us to utilize a simpler, univariate approach to answer our questions. Future studies could consider multivariate approaches to improve the predictability of any target species or assemblage.

## 4 CONCLUSIONS

The dynamics of individual phytoplankton taxa are noisy and typically difficult to predict. We used empirical dynamic modeling to assess the univariate predictability of taxa and assemblage (i.e., groups of more than one taxa) dynamics over monthly timescales and found that increasing the number of taxa in an assemblage increased predictability. Predictability was also significantly affected by how species were grouped together. Dinoflagellates and large phytoplankton (>12 μm cell radius) had lower overall predictability and did not increase in predictability with assemblage size. In contrast, aggregating species as coccolithophores, diatoms, and phytoflagellates led to improved predictability of the composite assemblage abundance time series over individual taxa. Similarly, small phytoplankton (<5 μm cell radius) were more predictable in assemblages than as individual taxa and this predictability exceeded that which we expect from seasonality alone. The presence of noise in our simulations, such as observational error and stochastic environmental influence, reduced the overall predictability of phytoplankton taxa.

While our study considers only 22 years of data from one temperate coastal ocean location, our findings have several implications. Firstly, field monitoring programs should continue to focus efforts on species‐level identification of dinoflagellates and large phytoplankton. In contrast, high predictability of smaller phytoplankton and coccolithophores, diatoms, and phytoflagellates could be achieved by aggregating them together, for example, by size fractionating measurements or identifying species only to genus or higher level. Overall, our results suggest that the dynamics of species assemblages will often be more predictable than that of individual species, but that the increase with assemblage size depends upon how species are grouped together. In certain cases, forecasting “portfolios” or assemblages of species together rather than as individuals may improve population forecasts and the management of living marine resources.

One such example of how our results may influence management of living marine resources is the food available for forage fish such as sardines and anchovies in coastal upwelling biomes (Checkley et al., 2017). While individual species fed upon by these economically important fish might not be individually predictable, aggregating across prey species within the preferred prey size range of each fish species might increase predictability of total available food and facilitate better management of the fisheries, especially by accounting for variability in predator responses and predator–prey interactions (Hunsicker et al., 2011). Another example is forecasting of phytoplankton genera (e.g., Anderson et al., 2016). The diatom genus *Pseudo*‐*nitzschia*, for example, includes many species and a range of strains within species (Hubbard et al., 2008; Trainer et al., 2012). Certain strains and species of *Pseudo*‐*nitzschia* spp. produce domoic acid, a potent neurotoxin with negative impacts on ecosystems. Our results suggest it may be more feasible to predict the total abundance of *Pseudo*‐*nitzschia* spp. than constituent species and strains.

Future studies should assess whether similar increases in predictability with increasing assemblage size occur in other marine, aquatic, and terrestrial systems, and assess whether the implications of this study apply more broadly.

## CONFLICT OF INTEREST

The authors declare no conflicts of interest.

## AUTHOR CONTRIBUTIONS

**Vitul Agarwal:** Conceptualization (equal); Formal analysis (equal); Investigation (equal); Methodology (equal); Software (equal); Visualization (equal); Writing‐original draft (equal); Writing‐review & editing (equal). **Chase C. James:** Conceptualization (equal); Formal analysis (equal); Investigation (equal); Methodology (equal); Software (equal); Supervision (equal); Validation (equal); Visualization (equal); Writing‐review & editing (equal). **Claire E. Widdicombe:** Data curation (equal); Funding acquisition (equal); Resources (equal); Validation (equal); Writing‐review & editing (equal). **Andrew D. Barton:** Conceptualization (equal); Formal analysis (equal); Funding acquisition (equal); Investigation (equal); Methodology (equal); Project administration (equal); Supervision (equal); Validation (equal); Visualization (equal); Writing‐review & editing (equal).

## Supporting information

Appendix S1

## Data Availability

Up to date versions of L4 data are available from the British Oceanographic Data Centre (www.bodc.ac.uk). Part of the data we use (1992‐2009) can be found at https://doi.org/10.1594/PANGAEA.758061.

## APPENDIX 1

**TABLE A1:** Taxonomic classification (e.g., diatom, dinoflagellate, coccolithophore, or phytoflagellate), approximate cell radius (µm), mean abundance (cells L^−1^), and standard deviation in abundance (cells L^−1^) of all phytoplankton time series at L4 used in this study. The mean and standard deviation are calculated for each taxon over the entire time series (1992–2014) using monthly averaged data

Taxa	Classification	Approximate cell radius (µm)	Mean abundance (cells L^−1^)	Standard deviation (cells L^−1^)
*Achnanthes longipes*	Diatom	11.19	1.40	20.23
*Actinocyclus*	Diatom	15.33	5.85	19.03
*Actinoptychus senarius*	Diatom	14.64	6.51	22.58
*Asterionellopsis glacialis*	Diatom	5.05	7.13	64.64
*Bacillaria paxillifera*	Diatom	10.16	50.89	232.86
*Bacteriastrum furcatum*	Diatom	10.40	47.55	586.19
*Trigonium alternans*	Diatom	10.74	0.96	6.49
*Brockmanniella brockmannii*	Diatom	7.94	34.56	253.59
*Odontella mobiliensis*	Diatom	29.86	21.15	206.12
*Odontella sinensis*	Diatom	42.79	17.54	101.41
*Cerataulina pelagica*	Diatom	13.52	1702.15	8233.12
*Chaetoceros*	Diatom	2.12	2299.60	17621.18
*Chaetoceros affinis*	Diatom	3.88	582.81	3532.86
*Chaetoceros anastomosans*	Diatom	3.88	2.39	20.91
*Chaetoceros f. borealis*	Diatom	3.88	0.97	9.26
*Chaetoceros brevis*	Diatom	3.88	2.60	41.84
*Chaetoceros compressus*	Diatom	2.86	1640.22	8787.11
*Chaetoceros concavicornis*	Diatom	9.04	0.45	5.10
*Chaetoceros costatus*	Diatom	3.88	26.69	272.80
*Chaetoceros curvisetus*	Diatom	5.49	1754.33	13185.63
*Chaetoceros danicus*	Diatom	9.04	67.74	147.77
*Chaetoceros debilis*	Diatom	6.31	4851.58	42201.66
*Chaetoceros decipiens*	Diatom	7.54	395.98	1879.14
*Chaetoceros densus*	Diatom	10.40	148.25	542.64
*Chaetoceros didymus*	Diatom	5.49	493.04	2499.90
*Chaetoceros eibenii*	Diatom	10.40	25.39	186.93
*Chaetoceros externus*	Diatom	3.88	0.16	2.49
*Chaetoceros filiformis*	Diatom	2.43	23.53	200.76
*Chaetoceros fragilis*	Diatom	2.43	44.84	612.20
*Chaetoceros laciniosus*	Diatom	3.88	90.39	372.51
*Chaetoceros lauderi*	Diatom	9.04	20.86	134.19
*Chaetoceros peruvianus*	Diatom	6.21	10.54	113.61
*Chaetoceros protuberans*	Diatom	5.49	204.05	2054.18
*Chaetoceros radicans*	Diatom	2.90	1837.16	15132.61
*Chaetoceros* resting spores	Diatom	2.82	1.61	18.48
*Attheya septentrionalis*	Diatom	1.68	229.53	2736.24
*Chaetoceros similis*	Diatom	1.68	11392.91	112310.28
*Chaetoceros simplex*	Diatom	2.53	2697.29	17484.47
*Chaetoceros socialis*	Diatom	2.90	24349.63	185351.44
*Chaetoceros teres*	Diatom	13.52	122.88	932.39
*Chaetoceros tortissimus*	Diatom	2.90	30.54	237.59
*Chaetoceros wighamii*	Diatom	2.90	669.54	10362.88
*Chaetoceros willei*	Diatom	3.88	11.61	152.13
*Corethron pennatum*	Diatom	16.97	15.69	40.15
*Coscinodiscus asteromphalus*	Diatom	30.42	0.38	2.51
*Coscinodiscus centralis*	Diatom	43.87	1.58	7.96
*Coscinodiscus concinnus*	Diatom	84.36	1.07	3.83
*Coscinodiscus granii*	Diatom	43.87	1.14	6.42
*Coscinodiscus radiatus*	Diatom	21.64	4.16	13.02
*Coscinodiscus wailesii*	Diatom	137.55	1.37	5.27
*Dactyliosolen blavyanus*	Diatom	31.08	3.91	39.79
*Dactyliosolen fragilissimus*	Diatom	11.38	1811.89	14391.04
*Delphineis*	Diatom	4.92	281.95	784.33
*Detonula pumila*	Diatom	13.29	503.97	3235.36
*Diploneis crabro*	Diatom	8.27	41.54	88.27
*Ditylum brightwellii*	Diatom	18.67	30.98	90.36
*Ephemera planamembranacea*	Diatom	19.81	0.06	0.69
*Eucampia zodiacus*	Diatom	10.37	671.43	2086.58
*Fragilaria*	Diatom	2.21	41.93	217.11
*Fragilariopsis*	Diatom	2.99	8.42	83.67
*Grammatophora*	Diatom	12.26	0.21	1.66
*Guinardia delicatula*	Diatom	11.38	5462.38	15906.88
*Guinardia flaccida*	Diatom	41.28	93.75	330.63
*Haslea wawrikae*	Diatom	4.75	26.72	266.88
*Lauderia annulata*	Diatom	15.00	1054.50	6863.94
*Leptocylindrus danicus*	Diatom	3.63	14681.86	51940.21
*Leptocylindrus mediterraneus*	Diatom	2.82	968.40	5663.51
*Leptocylindrus minimus*	Diatom	1.68	17475.65	80154.35
*Licmophora*	Diatom	13.52	1.68	12.61
*Lioloma delicatulum*	Diatom	4.56	16.41	121.64
*Lithodesmium undulatum*	Diatom	10.11	3.69	35.74
*Melosira*	Diatom	7.97	0.79	8.81
*Navicula*	Diatom	2.82	58.70	127.67
*Navicula distans*	Diatom	7.99	31.61	80.25
Small Pennate	Diatom	4.21	117.67	387.62
Pennate 30 µm	Diatom	5.60	308.74	3550.47
Pennate 50 µm	Diatom	5.60	45.29	174.33
V.small Pennate	Diatom	2.32	374.31	2863.01
*Nitzschia sigmoidea*	Diatom	3.69	26.70	52.95
*Cylindrotheca closterium*	Diatom	3.30	1486.41	2603.62
*Psammodictyon panduriforme*	Diatom	8.27	57.10	117.69
*Pseudo‐nitzschia delicatissima*	Diatom	2.32	30692.90	123034.66
*Pseudo‐nitzschia pungens*	Diatom	4.92	830.81	4075.22
*Pseudo‐nitzschia seriata*	Diatom	4.92	2668.65	13116.98
*Paralia sulcata*	Diatom	9.08	704.54	862.95
*Planktoniella sol*	Diatom	6.65	0.04	0.62
*Pleurosigma*	Diatom	13.52	88.49	127.63
*Pleurosigma planctonicum*	Diatom	27.03	21.73	86.42
*Podosira stelligera*	Diatom	25.01	16.54	31.86
*Proboscia alata*	Diatom	20.09	115.74	1103.47
*Proboscia alata* 5 µm	Diatom	11.20	1130.17	3756.49
*Proboscia truncata*	Diatom	37.00	10.04	34.90
*Rhizosolenia chunii*	Diatom	12.26	0.18	2.16
*Rhizosolenia hebetata f. semispina*	Diatom	17.79	21.44	103.41
*Neocalyptrella robusta*	Diatom	82.06	2.05	7.54
*Rhizosolenia setigera* 5 µm	Diatom	8.44	537.71	3724.33
*Rhizosolenia setigera* 25 µm	Diatom	28.62	85.54	787.80
*Rhizosolenia imbricata* 10 µm	Diatom	17.79	142.09	522.15
*Rhizosolenia imbricata* 5 µm	Diatom	11.20	149.30	520.27
*Rhizosolenia imbricata* 15 µm	Diatom	22.26	29.21	83.02
*Guinardia striata*	Diatom	7.78	901.08	5997.88
*Guinardia striata* (large)	Diatom	21.09	596.15	2587.05
*Rhizosolenia styliformis*	Diatom	37.00	10.30	25.02
*Proboscia alata (syn. f. gracillima)*	Diatom	7.20	10.63	78.38
*Roperia tesselata*	Diatom	14.43	18.16	36.30
*Skeletonema costatum*	Diatom	2.16	4328.23	32444.65
*Meuniera membranacea*	Diatom	14.92	458.66	1701.60
*Stephanopyxis palmeriana*	Diatom	29.25	9.23	58.85
*Helicotheca tamesis*	Diatom	11.28	3.29	20.79
*Thalassionema nitzschioides*	Diatom	3.50	1347.56	12205.66
*Thalassiosira punctigera*	Diatom	21.64	16.60	62.18
*Thalassiosira cf angulata*	Diatom	15.67	14.64	61.77
*Thalassiosira eccentrica*	Diatom	21.64	9.87	28.64
*Thalassiosira anguste‐lineata*	Diatom	13.52	59.46	517.67
*Thalassiosira gravida*	Diatom	13.63	139.95	1203.27
*Thalassiosira gravida* 15 µm	Diatom	7.51	97.99	804.72
*Thalassiosira rotula*	Diatom	13.52	417.75	1686.71
*Thalassiosira subtilis*	Diatom	8.59	637.34	7426.91
*Thalassiosira* 2 µm	Diatom	0.89	1255.11	6855.15
*Thalassiosira* 4 µm	Diatom	1.46	7169.26	46135.10
*Thalassiosira* 5 µm	Diatom	2.42	2470.67	24933.98
*Thalassiosira* 10 µm	Diatom	4.56	1925.30	9895.23
*Thalassiosira* 20 µm	Diatom	9.08	417.70	1872.42
*Thalassiosira* 30 µm	Diatom	13.63	97.26	607.67
*Thalassiosira* 40 µm	Diatom	14.43	87.20	1038.54
*Thalassiosira* 60 µm	Diatom	21.64	2.13	8.98
*Thalassiothrix*	Diatom	7.25	1.36	10.65
*Tropidoneis*	Diatom	7.09	30.80	106.30
*Nanoneis hasleae*	Diatom	1.19	776.36	4629.57
Undetermined Diatom	Diatom	2.21	0.93	9.99
*Amylax triacantha*	Dinoflagellate	15.00	2.97	19.94
*Neoceratium furca*	Dinoflagellate	20.10	8.53	50.17
*Neoceratium fusus*	Dinoflagellate	13.37	69.32	198.34
*Ceratium hexacanthum*	Dinoflagellate	22.86	0.05	0.83
*Neoceratium horridum*	Dinoflagellate	22.86	5.72	14.98
*Neoceratium lineatum*	Dinoflagellate	14.00	354.15	2397.98
*Ceratium longipes*	Dinoflagellate	22.86	0.85	3.69
*Neoceratium macroceros*	Dinoflagellate	22.86	1.67	9.12
*Neoceratium massiliense*	Dinoflagellate	23.88	0.20	1.24
*Neoceratium tripos*	Dinoflagellate	30.43	19.04	63.34
*Dinophysis acuminata*	Dinoflagellate	16.74	73.80	180.28
*Dinophysis acuta*	Dinoflagellate	24.67	27.81	220.20
*Dinophysis cf punctata*	Dinoflagellate	10.40	25.42	127.07
*Dinophysis sacculus*	Dinoflagellate	16.23	1.36	15.25
*Dinophysis tripos*	Dinoflagellate	24.67	1.02	4.30
*Karenia mikimotoi*	Dinoflagellate	10.00	11543.20	50183.10
*Gonyaulax*	Dinoflagellate	7.50	4.13	25.87
*Gonyaulax digitale*	Dinoflagellate	17.52	1.93	9.90
*Gonyaulax grindleyi*	Dinoflagellate	17.50	7.27	30.27
*Gonyaulax spinifera*	Dinoflagellate	16.23	31.54	141.32
*Alexandrium tamarense*	Dinoflagellate	12.50	1.75	13.10
*Gonyaulax verior*	Dinoflagellate	14.00	5.57	27.09
*Gymnodinium*	Dinoflagellate	4.93	39.64	138.35
*Gymnodinium pygmaeum*	Dinoflagellate	9.17	303.20	2392.93
*Heterocapsa*	Dinoflagellate	2.93	4779.12	33500.67
*Heterocapsa niei*	Dinoflagellate	2.93	1212.99	6825.67
*Heterocapsa triquetra*	Dinoflagellate	5.00	0.52	3.92
*Mesoporos perforatus*	Dinoflagellate	11.00	188.93	467.73
*Micranthodinium*	Dinoflagellate	6.30	56.08	177.54
*Prorocentrum balticum*	Dinoflagellate	4.47	10389.50	120772.43
*Tryblionella compressa*	Dinoflagellate	11.45	0.06	0.75
*Prorocentrum dentatum*	Dinoflagellate	3.13	200.76	2243.86
*Prorocentrum micans*	Dinoflagellate	14.12	383.68	1839.70
*Prorocentrum cordatum*	Dinoflagellate	3.21	28020.53	177643.32
*Prorcentrum triestinum*	Dinoflagellate	11.30	132.46	937.73
*Scrippsiella trochoidea*	Dinoflagellate	8.88	1181.40	4474.84
*Scrippsiella* (cyst)	Dinoflagellate	8.89	3.87	22.45
Unidentified Coccolithophorid	Coccolithophore	5.00	87.64	567.82
Holococcolithophorid 14 µm	Coccolithophore	5.00	210.73	1733.06
Holococcolithophorid 8 µm	Coccolithophore	2.98	861.91	5263.35
*Acanthoica quattrospina*	Coccolithophore	4.00	265.45	880.77
*Calciosolenia brasiliensis*	Coccolithophore	3.34	1.56	17.05
*Braarudosphaera bigelowii*	Coccolithophore	8.00	5.96	60.11
*Calyptrosphaera*	Coccolithophore	7.50	249.57	1742.70
*Coccolithus pelagicus*	Coccolithophore	7.00	7.60	40.94
*Coccolithus pelagicus f. hyalinus*	Coccolithophore	7.00	10.03	77.29
*Emiliania huxleyi*	Coccolithophore	2.52	61959.76	163719.83
*Coronosphaera*	Coccolithophore	4.00	86.41	1102.52
*Syracosphaera molischii*	Coccolithophore	2.98	24.05	178.77
*Gephyrocapsa*	Coccolithophore	2.98	311.91	1595.55
*Rhabdolithes claviger*	Coccolithophore	2.52	10.00	107.47
*Syracosphaera pulchra*	Coccolithophore	6.30	3.10	30.77
*Umbellosphaera*	Coccolithophore	5.00	201.71	1040.80
Flagellate 2 µm	Phytoflagellate	0.98	1655157.68	812729.13
Flagellate 5 µm	Phytoflagellate	2.49	667894.54	436517.09
Flagellate 15 µm	Phytoflagellate	4.93	16056.25	30492.76
*Corymbellus aureus*	Phytoflagellate	3.30	791.40	7001.26
*Cryptomonadaceae*	Phytoflagellate	2.52	72546.98	79015.08
*Dictyocha speculum*	Phytoflagellate	6.08	94.39	199.37
*Dictyocha fibula*	Phytoflagellate	6.08	20.84	132.27
*Dinobryon*	Phytoflagellate	2.29	4754.57	55343.02
*Eutreptiella*	Phytoflagellate	7.11	2485.47	33534.45
*Halosphaeria*	Phytoflagellate	49.17	4.41	28.25
*Meringosphaera*	Phytoflagellate	2.29	14398.94	228270.01
*Pterosperma*	Phytoflagellate	10.00	469.09	3351.76
*Pyramimonas*	Phytoflagellate	4.00	20.48	149.46
*Raphidophyceae*	Phytoflagellate	3.03	801.74	11609.17
*Phaeocystis*	Phaeocystis	2.99	93617.86	369305
