# Supplementary material for: Intraseasonal predictability of natural phytoplankton population dynamics
Source: Ecol Evol. 2021 Oct 28;11(22):15720–39. doi: 10.1002/ece3.8234 (PMC8601889; doi:10.1002/ece3.8234)
Supplement: Supplementary file 1 — Appendix S1 [file ECE3-11-15720-s001.docx]

**Appendix**

**Figure S1**: Abundance (cells L^-1^) of three noise model taxa with various levels of observational error: (A) No noise (B) Low Noise and (C) High noise. The values for the parameters can be found in Table 1.

**Figure S2**: Abundance (cells L^-1^) of three phytoplankton community model taxa (A) with no noise and inter-species interactions (B) with inter-species interactions and (C) with process noise. The values for the parameters can be found in Table 2.


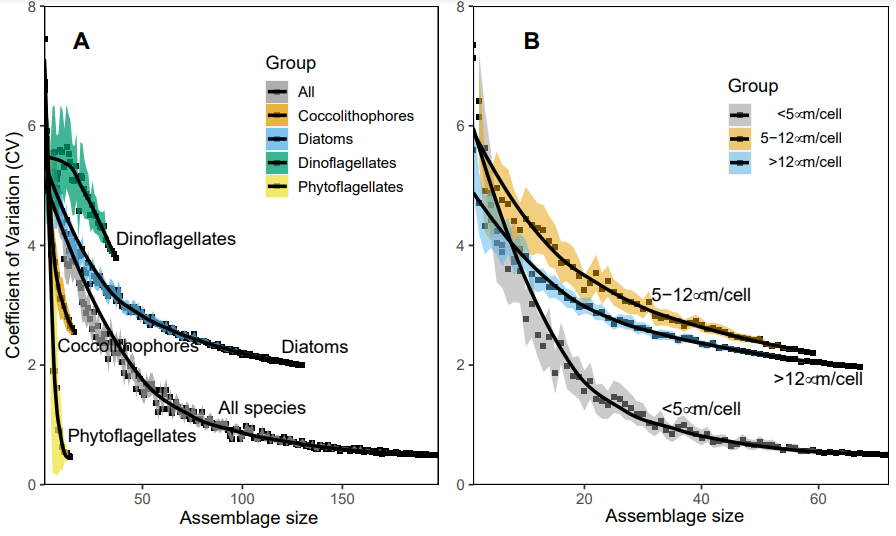


**Figure S3**: Coefficient of variation (CV) for assemblages of taxa based on taxa or/size-based classification. Each point is the mean of 1000 trials, and the black lines represent local regression fits for the data). The shaded regions are 95% confidence intervals (defined as $\pm1.96\times SE$).


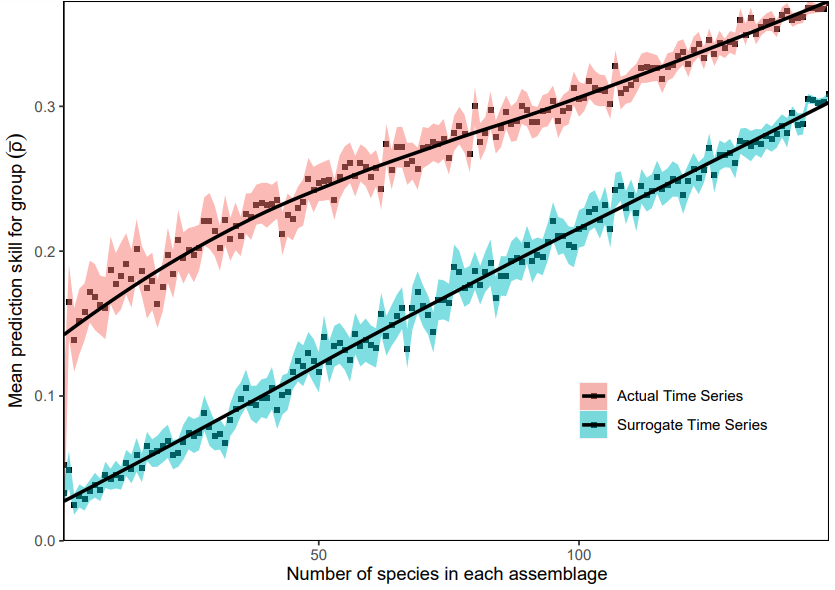


**Figure S4**: Mean prediction skill ($\bar{\rho}$) for assemblages of taxa (red) and their seasonal surrogates (blue), after removing the top 25% of most abundant taxa from the analysis. Each point is the mean of 100 trials, and the black lines represent local regression fits for both sets of data (actual time series and surrogates). The shaded regions are 95% confidence intervals (defined as $\pm1.96\times SE$). This figure is analogous to Figure 2, but with the most abundant taxa removed.


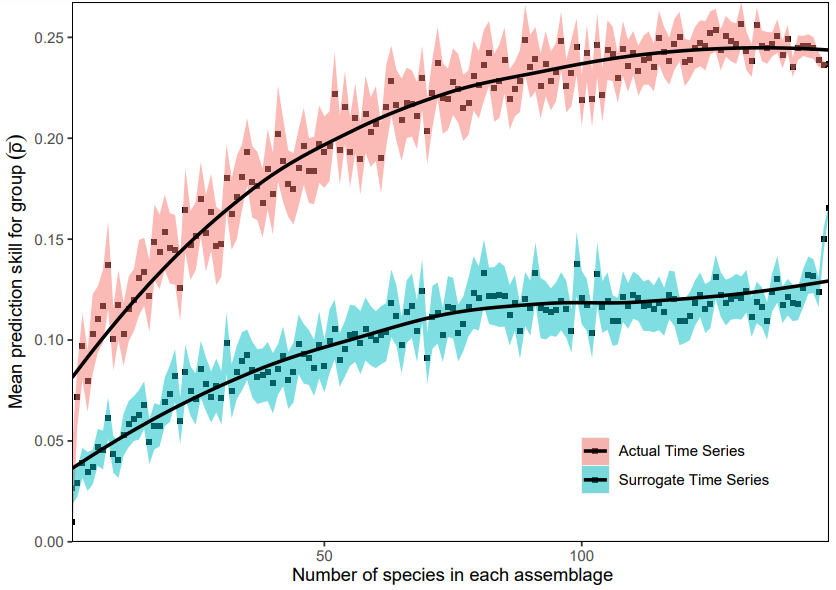


**Figure S5**: Mean prediction skill ($\bar{\rho}$) for assemblages of taxa (red) and their seasonal surrogates (blue), after removing the top 25% of most predictable taxa from the analysis. Each point is the mean of 100 trials, and the black lines represent local regression fits for both sets of data (actual time series and surrogates). The shaded regions are 95% confidence intervals (defined as $\pm1.96\times SE$). This figure is analogous to Figure 2, but with the most predictable taxa removed.
